# iNOS expressing macrophages co-localize with nitrotyrosine staining after myocardial infarction in humans

**DOI:** 10.3389/fcvm.2023.1104019

**Published:** 2023-03-30

**Authors:** Verena Wilmes, Ivan M. Kur, Andreas Weigert, Marcel A. Verhoff, Elise Gradhand, Silke Kauferstein

**Affiliations:** ^1^ Institute of Legal Medicine, University Hospital Frankfurt, Goethe University, Frankfurt am Main, Germany; ^2^ Institute of Biochemistry I, Faculty of Medicine, Goethe University, Frankfurt am Main, Germany; ^3^ Cardio-Pulmonary Institute (CPI), Goethe University, Frankfurt am Main, Germany; ^4^ Dr. Senckenberg Institute of Pathology, University Hospital Frankfurt, Goethe University, Frankfurt am Main, Germany

**Keywords:** iNOS, iNOS expressing macrophages, oxidative stress, myocardial Infarction, macrophage populations, inflammation

## Abstract

**Introduction:**

Inducible nitric oxide synthase (iNOS) produces micromolar amounts of nitric oxide (NO) upon the right stimuli, whose further reactions can lead to oxidative stress. In murine models of myocardial infarction (MI), iNOS is known to be expressed in infiltrating macrophages, which at early onset enter the infarcted zone and are associated with inflammation. In contrast cardiac tissue resident macrophages are thought to enhance regeneration of tissue injury and re-establish homeostasis. Both detrimental and beneficial effects of iNOS have been described, still the role of iNOS in MI is not fully understood. Our aim was to examine cell expression patterns of iNOS and nitrotyrosine (NT) production in human MI.

**Material and Methods:**

We examined in postmortem human MI hearts the iNOS mRNA expression by means of qPCR. Further we performed immunohistochemical stainings for cell type identification. Afterwards a distance analysis between iNOS and NT was carried out to determine causality between iNOS and NT production.

**Results:**

iNOS mRNA expression was significantly increased in infarcted regions of human MI hearts and iNOS protein expression was detected in resident macrophages in infarcted human hearts as well as in controls hearts, being higher in resident macrophages in MI hearts compared to control. Furthermore in MI and in healthy human hearts cells showing signs of NT production peaked within 10–15 µm proximity of iNOS+ cells.

**Discussion:**

These results indicate that, unexpectedly, resident macrophages are the main source of iNOS expression in postmortem human MI hearts. The peak of NT positive cells within 10–15 µm of iNOS+ cells suggest an iNOS dependent level of NT and therefore iNOS dependent oxidative stress. Our results contribute to understanding the role of iNOS in human MI.

## 1. Introduction

Inducible nitric oxide synthase (iNOS) is one of the three NOS isoforms, which produce nitric oxide (NO). In contrast to the constitutive forms of neuronal (nNOS) and endothelial nitric oxide synthases (eNOS) iNOS produces not only nanomolar concentrations of NO for seconds or minutes but it generates micromolar amounts of NO for hours or even days upon activation (1). The cellular functions of NO may be concentration dependent (2). At low concentrations, NO causes smooth muscle relaxation, vasorelaxation and regulates blood flow, whereas at high concentrations NO can be cytotoxic and induce apoptosis (1, 3). Even more importantly, NO forms peroxynitrite by reacting with superoxide anion. The concentration of NO is a key regulator of peroxynitrite production, outcompeting the reaction of superoxide dismutase for superoxide anion, due to the burst of NO produced by iNOS (4). Peroxynitrite is a strong oxidant causing oxidative stress by lipid peroxidation and nitrosylation of proteins, having detrimental effects, e.g., to cardiomyocytes (1, 5–7). Furthermore peroxynitrite is described to inactivate anti-proteases (8) and to activate matrix-metalloproteinases, which are known to degrade and remodel the extracellular matrix under physiological and pathological conditions (9). Loss of myocardial extracellular matrix, which is driven by matrix metalloproteinases, is considered as one of the determinants of left ventricular remodeling after myocardial infarction (MI) (10). In the lung iNOS dependent NO production and subsequent peroxynitrite formation is suggested to promote alveolar destruction and emphysema (11).

iNOS is mainly induced by stimuli such as hypoxia and inflammation (12). Increased iNOS expression has been described in murine and rabbit models of MI (3, 13–16) as well as in our previous studies in post mortem human infarction hearts (17, 18). The cellular expression pattern of iNOS is mostly described in murine models of infarction and in these models iNOS seems to be predominantly expressed in macrophages but is also described in cardiomyocytes (3, 15, 19).

In the human myocardium, different subsets of macrophages are present (20–23), which are roughly divided into infiltrating (CD68+, CD206−, CD163−) and resident macrophages (CD68+, CD206+, CD163+), with CD68 being an antigen expressed by both subsets. Prenatally yolk-sac-derived tissue-resident macrophages regenerate themselves locally without contribution of blood monocytes. These resident cardiac macrophages express a large amount of the “M2-designated” markers (22). The functions of those tissue-resident macrophages differ and depend mainly on their microenvironment. However, it is known that they enhance regeneration after tissue injury and re-establish homeostasis (24). “M1” infiltrating macrophages, which comprise blood derived monocytes, express numerous pro-inflammatory markers (22) and are known to express iNOS in mice (20). Infiltrating macrophages enter the infarcted zone in the initial stage of MI for clearance of dead cells and matrix debris and cause a gradual enlargement of the infarcted zone of MI hearts (22). Even though inflammation is required for clearance of dead cells and tissue regeneration, an exaggerated inflammatory reaction can delay the healing process of the myocardium (22). Even though iNOS is classically viewed as a marker of infiltrating macrophages (20, 21, 25), the limitations of this nomenclature are described (21, 26, 27).

Saini and Singh (28) describe iNOS expression in neutrophils and furthermore in cytokine stimulated macrophages, fibroblasts, vascular smooth muscle cells and hepatocytes, which confirms the observation of Neri et al. (29) that iNOS can be expressed in every cell upon the right stimuli. However, except for approaches in the study of Shimojo et al. (30) the cellular expression pattern of iNOS in human MI hearts has not been described in detail.

Therefore, the aim of this study was to examine iNOS expression in infarction hearts, non-infarcted regions and healthy controls. Furthermore, the detection of nitrotyrosine (NT), the fingerprint of peroxynitrite in tissue, was evaluated and a distance analysis between iNOS and NT was performed to gain a first hint towards causality. We aim at further understanding the role of iNOS in the pathophysiology of MI by comparing expression patterns and parameters of oxidative stress in human MI hearts.

## 2. Material and methods

### 2.1. Sample selection

Cardiac tissue samples from deceased individuals were collected during court ordered autopsies at the Institute of Legal Medicine, University Hospital, Goethe University Frankfurt, Germany. The control group consisted of microscopically healthy, unharmed hearts from overall healthy individuals who died from accidents or committed suicide. Therefore, only individuals who died a non-natural death were included. The MI group consisted of hearts with macroscopically visible signs of cardiac infarction. All samples were histologically revised by an experienced pathologist and RNA was extracted. From the infarction zone of the MI hearts, two replicates were taken, either from posterior or anterior wall, depending on the infarct localization. Additionally, two samples from the macroscopically unaffected wall of the MI hearts and two from the anterior and posterior wall of control hearts were taken. Variations within one heart and the heterogeneity of the infarctions were considered by taking more than one tissue sample. All samples were obtained from the left ventricle.

The MI and the control group are shown in Tables 1, 2.

**Table 1:** Gender, mean Age (years), myocardial infarction (MI) age and mean postmortem interval (PMI, days) of the myocardial infarction group.

Gender Male/Female	Age	Acute/Older Infarction	PMI
21/6	68,6	17/10	6

**Table 2:** Gender, mean Age (years) and mean postmortem interval (PMI, days) of the control group.

Gender Male/Female	Age	PMI
8/2	40,5	8

### 2.2. Histological examination

Tissue samples were fixed in 4.5% buffered formalin, embedded in paraffin and sections of 5 µm were stained with haematoxylin-eosin.

### 2.3. Immunhistological examinations of iNOS, nitrotyrosine (NT) and Cd68

Paraffin-embedded heart samples were sectioned at 5 µm and after heating for 30 min at 60°C, deparaffinized with xylol and graded alcohol. Blocking of endogenous peroxidase was applied by incubation with 3% hydrogen peroxidase for 15 min. For antigen retrieval and to increase cell permeability for the antibody, pre-treatment was applied. Therefore, the samples were boiled in citratebuffer (0,1 M, pH = 6) three times for 1 min with 5 min breaks in between. Subsequently slides were incubated with 20% goat normal serum for 20 min at room temperature for blocking. Incubation with primary antibody anti-iNOS (NOS2 mouse monoclonal, Santa Cruz, CA, USA) diluted 1:50 in phosphate buffered saline (PBS) was applied over night at 4–8°C. Afterwards, the samples were incubated at room temperature for 30 min and washed twice with PBS. The utilized detection system was the LSAB2 system-HRP kit (Dako, Copenhagen, Denmark) an avidin-biotin technique in which the biotinylated secondary antibody reacts with several peroxidase-conjugated streptavidin molecules. The biotinylated secondary antibody was applied for 30 min at room temperature. After washing wish PBS twice, the samples were incubated with horseradish peroxidase (HRP) for 30 min at room temperature und washed twice again with PBS. Afterwards slides were incubated with AEC (Dako, Copenhagen, Denmark) for 15 min and washed with PBS. The samples were counterstained with haematoxylin for 3 min, dehydrated, cover slipped and examined with a transmission light microscope (Zeiss, Jena, Germany).

For NT staining, samples were deparaffinized at 60°C for 30 min and for antigen retrieval boiled for 40 min in EnVision^TM^ Flex Target Retrieval Solution Low pH (Dako, Copenhagen, Denmark). The primary antibody anti-nitrotyrosine (nitrotyrosine mouse monoclonal, Santa Cruz, CA, USA) was diluted 1:400 in antibody diluent (Dako, Copenhagen, Denmark) and applied on the samples for 30 min. Slides were washed with tris buffered saline (TBS) and the subsequently utilized detection system was the EnVision^TM^ Flex (Dako, Copenhagen, Denmark). Samples were incubated with hydrogen peroxidase for 5 min and washed with TBS. HRP was applied for 20 min and samples were washed in TBS again. Incubation with DAB (Dako, Copenhagen, Denmark), followed for 10 min. Every incubation step was applied at room temperature. After counterstaining with haematoxylin, samples were dehydrated, cover slipped and examined with a transmission light microscope (Zeiss, Jena, Germany).

For staining of CD68, samples were deparaffinized in a two-phase dewaxing procedure using Clearify^TM^ Clearing Agent (Dako, Copenhagen, Denmark). Antigen retrieval was applied using EnVision^TM^ Flex Target Retrieval Solution High pH at 97°C for 20 min. After incubation with washing buffer of the EnVision Flex^TM^ Kit (Dako, Copenhagen, Denmark) for 2,40 min, the primary antibody CD68 KP1 (EnVision Flex^TM^ mouse monoclonal, Dako, Copenhagen, Denmark) was applied for 20 min. After another washing step for 2 min, slides were incubated with peroxidase for 3 min. Subsequently samples were washed again for 2 min and incubated for 20 min with HRP. After several rounds of washing, samples were incubated 5 min with DAB (Dako, Copenhagen, Denmark). After counterstaining with haematoxylin, samples were dehydrated, cover slipped and examined with a transmission light microscope (Zeiss, Jena, Germany).

For semiquantitative analysis of iNOS, NT and CD68 staining, samples were scored in a double-blind manner. The intensity of immunopositive expression was assessed on a scale of 0–3 as follows: only perivascular staining (1), perivascular and isolated tissue staining (2) and pronounced perivascular and tissue staining (3). Quantity of staining was indicated by:+(few), normal (++) and strong (+++). For analysis of nitrotyrosine, staining intensity was evaluated only as quantity.

### 2.4. Tissue preparation and immunostaining

Tissue sections were deparaffinized by 1 h incubation at 60°C. Tissue sections (5 µm) were stained with Opal 7-Color Automation immunohistochemistry Kits (Akoya Bioscience) in the BOND-RX Multiplex IHC Stainer (Leica). The first step of the automated IHC included a fixation step with 4% Formalin (Roth, P087.5) in PBS. Each section was put through 6 sequential rounds of staining, which included blocking in 5% BSA, followed by incubation with primary antibodies of the following panel (NT, Santa Cruz, sc-32757; NOS2, Santa Cruz, sc-7271; CD163, Abcam, ab182422; CD206, Cell signaling, 91992S; CD68, DAKO, M0876), corresponding secondary HRP-conjugated antibodies and Opal fluorophores as described. Nuclei were counterstained with 4′,6-diamidino-2-phenylindole (DAPI) contained in the Opal 7-Color Automation IHC Kits, and slides were mounted with Fluoromount-G (SouthernBiotech).

### 2.5. Microscopy and image analysis

Imaging was performed with the VectraPolaris imaging system (Akoya Bioscience), and images were analyzed by using the phenotyping application of the inForm software V2.4.10 (Akoya Bioscience). Phenotype classification was performed automatically by the written algorithm using inform Software. Multispectral image analysis was performed with Phenochart, InForm Image analysis software (Akoya Biosciences Inc.), and HALO software (Indica labs). For distance analysis in HALO software 5 slides of the infarcted regions, 5 slides of the non-infarcted regions and 5 healthy controls were examined, calculating proximity distances from one population to the other and vice-versa in a delimited radius range.

### 2.6. Reverse transcription quantitative polymerase chain reaction

RNA extraction, cDNA synthesis and quantitative real-time PCR (qPCR) was carried out as previously described (18). In short, one hundred milligrams of cardiac tissue were mechanically homogenized in 1 ml Trizol. Genomic DNA digestion was performed and subsequently extracted RNA samples were purified. For synthesizing single stranded cDNA random priming conditions were used. Quantitative real time PCR was performed using the following Taq Man® Gene Expression Assays: inducible nitric oxide synthase (NOS2, amplicon size 67 bp, assay ID: Hs01075529_m1, refseq.-nr.: NM_000625.4). Peptidylprolyl isomerase A (PPIA, amplicon size 98 bp, assay ID: Hs99999904_m1, refseq.-nr.: NM_021130.4), TATA-box binding protein (TBP, amplicon size 91 bp, assay ID: Hs00427620_m1, refseq.-nr.: NM_003194.4) and tumor protein translationally controlled 1 (TPT1, amplicon size 131 bp, assay ID: Hs02621289_g1, refseq.-nr.: NM_003295.3) (Applied Biosystems, Darmstadt, Germany) were used as endogenous reference genes due to their postmortem stability and their stable expression in cardiac tissue, as previously described (31, 32). For each sample triplicates were applied as well as no template negative controls for each assay.

Analysing the qPCR efficiency was performed by using LinReg PCR Software (33). The qPCR was efficient when the values were between 1,8 and 2,0. Values lower than 1,8 indicate an inadequate efficacy and were excluded. Efficacy values per heart assay were determined and used to correct the corresponding Cq values.

For qPCR of iNOS 200 ng cDNA were used. After reverse transcription cDNA concentrations were not measured, since the reverse transcription ratio of RNA:cDNA is 1:1.

### 2.7. Data analysis

qPCR was carried out by using the Software 7500, version 2.0.6 and Data Assist v3.01 served for analyzing the qPCR raw data. Calculation of fold change was carried out using the *ΔΔ*Cq method (34).

The software R version 3.5.1 was used for statistical analysis. Linear mixed effects model and pairwise *post hoc* comparisons were applied and significance was adjusted, by using Tukey's multiple comparisons test. Two individual measurements of the unaffected and the affected regions of MI hearts were included in the statistical analysis to detect local differences in the gene expression per heart and two of the anterior and posterior wall for each control heart.

## 3. Results

### 3.1. Sample selection

Histological examinations confirmed the macroscopic pathological findings of MI. Acute, subacute infarctions or scar areas were present, either focal or diffuse. The non-infarcted areas revealed no cellular changes and no obvious cardiomyocyte injuries. In some cases, mild diffuse or localized scaring after older myocardial hypoxia was observed. The control group did not show any histopathological cardiac changes.

### 3.2. Increased iNOS mRNA expression in MI hearts

**Figure 1:**
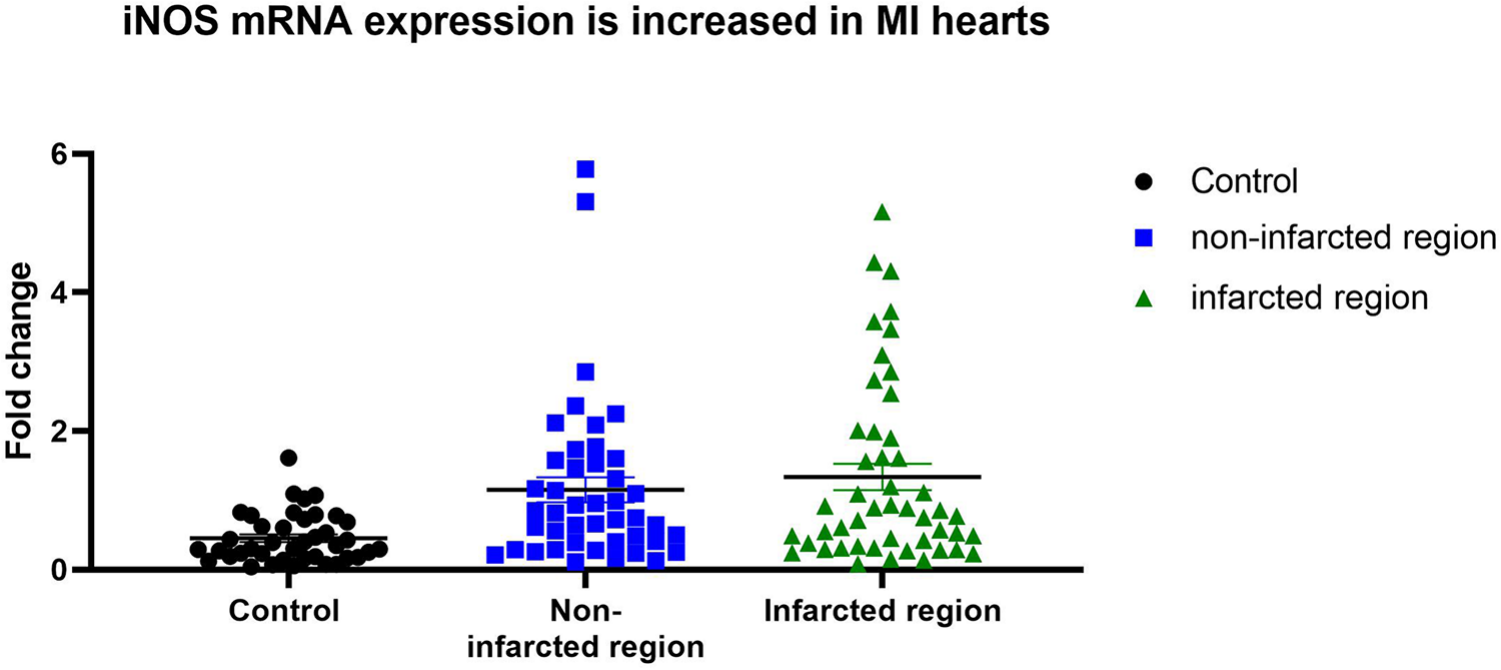
iNOS expression in controls, non-infarcted and infarcted regions. iNOS mRNA expression level in control hearts (*n* = 10), in non-infarcted (*n* = 23) and in infarcted (*n* = 25) regions of MI hearts were determined by qPCR. Individual values were plotted and show significant upregulation of iNOS expression in infarcted regions in comparison to controls Linear mixed effects model was applied and significance was adjusted after normality by using Tukey's multiple comparisons test.

iNOS expression in control hearts, as well as in non-affected and affected regions of MI hearts is shown in Figure 1. In infarcted regions the iNOS expression was significantly increased (*p* = 0,01) in comparison to the control group. Non-infarcted regions of MI hearts exhibited a strong tendency towards increased iNOS expression, albeit not reaching statistical significance (*p* = 0,0523) in comparison to the control hearts.

### 3.3. Increased expression of iNOS, nitrotyrosine (NT) and Cd68± macrophages in MI hearts

In Table 3 the mean IHC staining intensities for iNOS, CD68 and NT in each group are shown. The iNOS protein expression was strongly increased in non-infarcted and infarcted areas in comparison to healthy control myocardium. NT was also strongly visible in non-infarcted and infarcted areas in comparison to control tissue. No difference in NT staining was found between acute and older infarctions.

**Table 3:** Mean immunhistological (IHC) staining intensities for iNOS, CD68 and NT in the controls, non-infarcted and infarcted regions. The intensity of immunopositivity was assessed on a scale of 0–3 as follows: only perivascular staining (1), perivascular and isolated tissue staining (2) and pronounced perivascular and tissue staining (3). Quantity of staining was indicated by:+(few), normal (++) and strong (+++).

	Control group	Non-infarcted areas	Infarction areas
iNOS	1++	2+	2++
CD68	1++	2+++	2+++
NT	+	++	++

### 3.4. Cd68± macrophages show iNOS expression in human MI hearts

**Figure 2:**
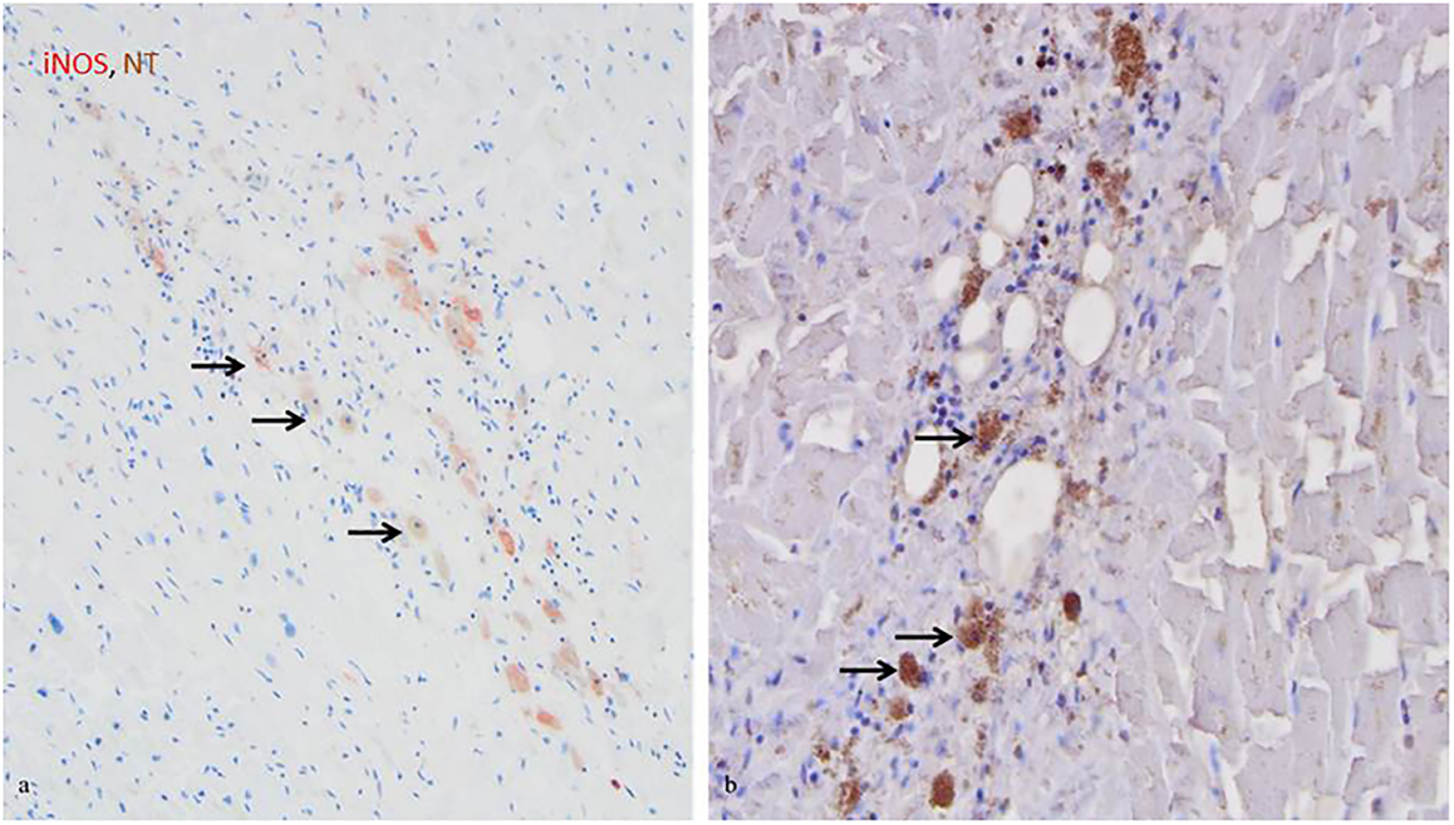
iNOS and NT co-expression by CD68+ macrophages in infarcted regions. (**A**) iNOS expression by macrophages in the acutely infarcted tissue, as indicated by the arrows (red, 20x). (**B**) NT expression by macrophages in the acutely infarcted tissue, as indicated by the arrows (brown, 20x). Cell nuclei were counterstained with haematoxylin.

Next, the cellular expression pattern of iNOS was evaluated by comparison of CD68+ and iNOS expressing cells. In control hearts iNOS protein and CD68 macrophages were restricted to the perivascular tissue and only rarely visible within the myocardium. In the infarcted and non-infarcted regions, an upregulation of iNOS protein expression and an increase of CD68+ macrophages in comparison to healthy control tissue was observed, indicating an accumulation of both in infarcted hearts (Table 3). Furthermore, the CD68+ macrophages were detected as the main iNOS expressing cells in human MI hearts, in the infarcted, as well as the non-infarcted areas (Figure 2). However, more CD68+ cells than iNOS expressing cells were observed.

Of note, in a few non-infarcted regions, where microscopical examinations revealed no cellular changes, only acute hypoxia or smallest fibrotic changes, a strong increase in iNOS and CD68 protein levels was observed (Table 3).

### 3.5. The presence of iNOS and NT in macrophages indicates oxidative stress in MI hearts

In the infarcted and non-infarcted areas, a strong NT staining was observed, while in control hearts no or only weak staining of NT was observed (Table 3). NT was mainly found in cardiomyocytes and to a lesser extent in CD68+ macrophages, indicating co-staining of iNOS and NT (Figure 2).

In comparison to acute and older infarctions, a reduced iNOS protein staining was detected in older infarctions. Yet, the intensity of the NT staining showed no difference between older and acute infarctions (data not shown).

### 3.6. Infiltrating macrophages express less iNOS compared to resident macrophages in human hearts

**Figure 3:**
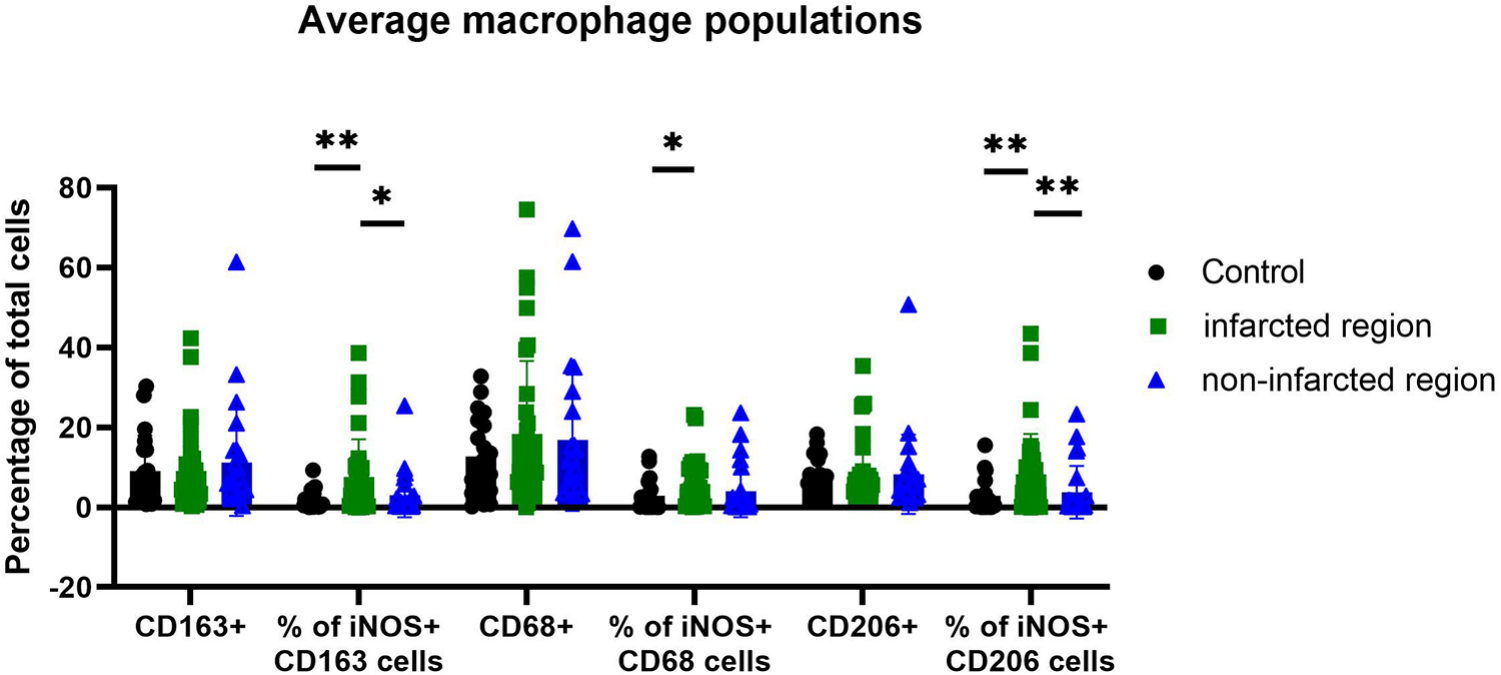
Amount of iNOS-producing macrophage subpopulation in controls, non-infarcted and infarcted regions. Individual values were grouped and plotted. Statistical significance was indicated after normality and Mann-Whitney tests. Significance from left to right: % of iNOS+ CD163 cells (*p*-value 0,0069 and 0,0116); % of iNOS+ CD68 cells (*p*-value 0,0284) and % of iNOS+ CD206 cells (*p*-value 0,0045 and 0,0060). In CD163+ and CD206+ cells iNOS expression is stronger increased than in CD68+ cells.

**Figure 4:**
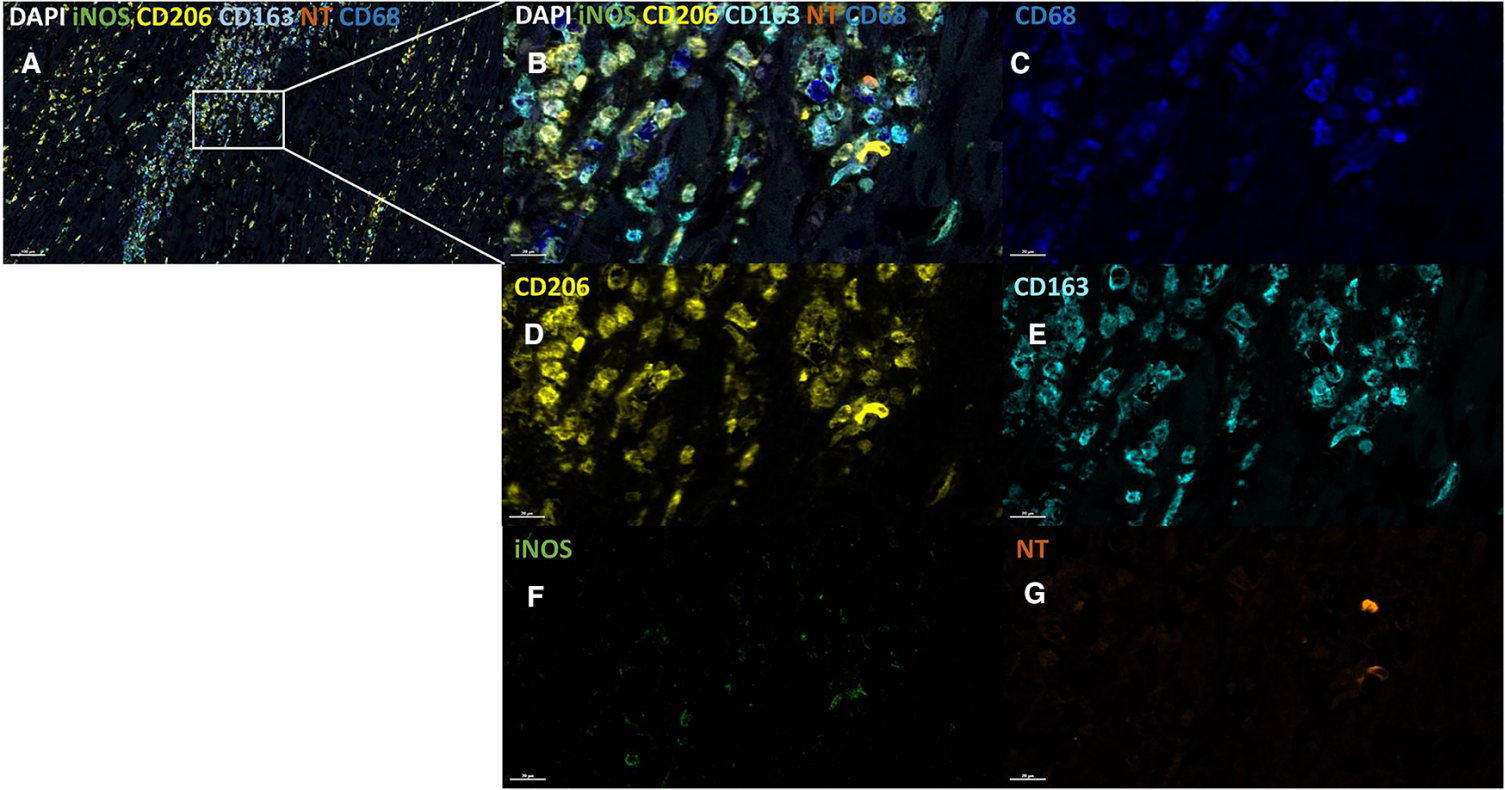
iNOS expression and NT production in different macrophage populations in infarcted regions. Infarcted tissue section, low magnification (**A**) and high magnification (**B**) stained using the following markers: CD68 (dark blue, **C**), CD206 (yellow, **D**), CD163 (light blue, **E**), iNOS (green, **F**) and NT (orange, **G**). iNOS is visible within the CD163+ and CD206+ macrophage. Cell counterstain was performed with DAPI (white). Scale bars are shown in the images.

For further exploration of iNOS expressing macrophages in human MI hearts and healthy control hearts the BOND-RX Multiplex Stainer system was used, with antibodies against resident vs. infiltrating macrophage markers. The results showed iNOS expression mainly in CD206+ and CD163+ macrophages, which are mostly resident macrophages, and to a lesser extent in CD68+ macrophages, indicating that infiltrating macrophages did not express significantly higher iNOS levels (Figures 3, 4). Staining was performed in the infarcted regions, the non-infarcted regions and the control hearts.

**Figure 5:**
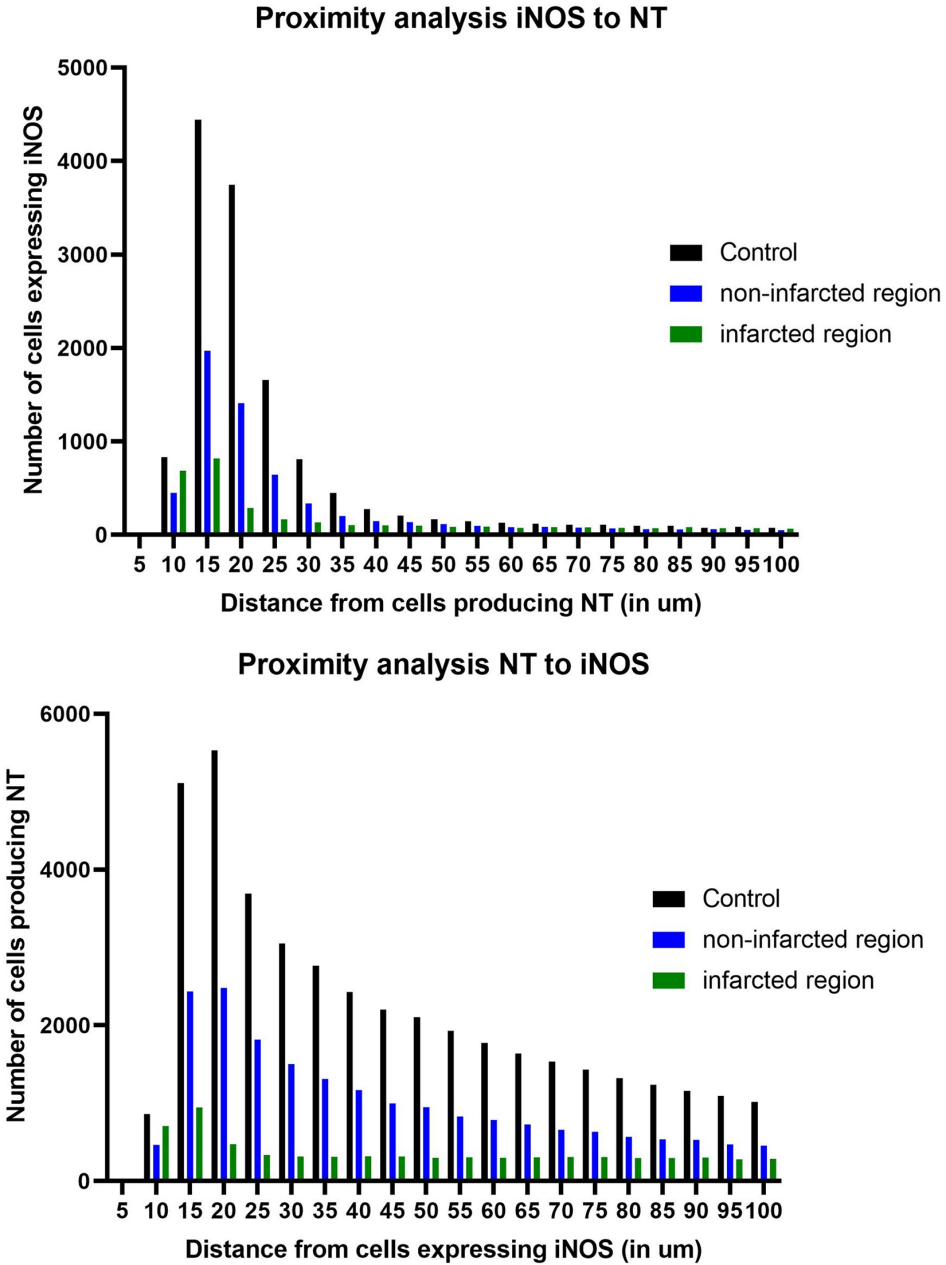
Distance between iNOS+ and nitrotyrosine+ (NT+) cells in controls, non-infarcted and infarcted regions. (**A**) Number of iNOS+ and NT+ cells within 100 µm in non-infarcted regions of MI hearts. (**B**) Number of NT+ and iNOS+ cells within 100 µm in infarcted regions of MI hearts. NT+ cells peak within a distance of 10–15 µm of iNOS.

Even though most macrophages in the heart were CD68+, the percentage of all iNOS expressing CD68+ macrophages did only slightly increase in MI hearts in comparison to control hearts. However, the amount of iNOS expression specifically in CD206+ and CD163+ macrophages did markedly increase in MI hearts, in comparison to control hearts. Therefore, the increase in iNOS protein expression in human MI hearts is due to an increase of iNOS production in CD206+ and CD163+ macrophages. Interestingly, the amount of CD206+ and CD163+ macrophages did not or only slightly increase in the MI hearts in comparison to control hearts, supporting the notion that these cells comprise the resident subset. However, there was an increase of all CD68+ macrophages in MI hearts in comparison to healthy hearts, indicating an influx of newly recruited monocytes/macrophages as expected upon myocardial infarction.

### 3.7. Nt production occurs in the vicinity of iNOS± cells

NT production increased in the infarcted and non-infarcted regions in comparison to healthy controls. Since iNOS is a known contributor to peroxynitrite emergence and subsequent NT production, a distance analysis was applied, to examine potential causality between iNOS expression and NT production. In five infarcted regions, five non-infarcted regions and five healthy controls, the distance of iNOS+ and NT+ cells were measured in a radius of 100 µm (Figure 5). In all groups there was a peak of cells within 10–15 µm, demonstrating the close proximity between NT and iNOS expressing cells. When the analysis was run from iNOS to NT, we observed a higher number of cells expressing iNOS towards cells producing NT within 10–15 µm.

## 4. Discussion

### 4.1. Resident macrophages are the main source of iNOS in human MI hearts

A significant increase in iNOS mRNA expression in MI hearts was found, together with a strong increase of iNOS protein expression in infarcted and non-infarcted regions compared to control hearts. Our results indicate that resident CD206+ and CD163+ macrophages, expressing classical markers of “M2” macrophages (22), are the main source of iNOS expression in human MI hearts. The findings of Gredic et al. (26) of CD206+ and iNOS+ macrophages in close proximity of remodeled vessels in lungs of human COPD patients support our observations. This is crucial since iNOS is considered a marker of pro-inflammatory, infiltrating macrophages (20, 25) and resident macrophages are thought to enhance tissue regeneration and re-establish homeostasis (24).

Moreover, Gredic et al. (26) described an increase in CD206+ resident macrophages, when wild type mice expressing iNOS were exposed to tobacco-smoke. This increase was abolished in iNOS knockout mice. Even more interesting they found evidence for an iNOS-dependent crosstalk between resident macrophages and pulmonary artery smooth muscle cells (PASMCs) which drives proliferation of these vascular cells and therefore, pulmonary vascular remodeling upon smoke-exposure, a pathway that can be prevented by deletion of resident macrophage derived iNOS. They hypothesized, that this regulatory role of iNOS is unique for specific conditions, including smoke-exposure and communication between macrophages and PASMCs. These results may apply to the current study in human MI hearts, since smoke exposure resembles hypoxic injury in MI, and we also found increased iNOS production in CD206+ and CD163+ macrophages. Although in the current study we did not examine crosstalk between cardiac macrophages and other cardiac cells, this is a known phenomenon. Ramanujam et al. (35) described miR-21 dependent crosstalk between inflammatory, infiltrating macrophages and fibroblasts in a murine pressure overload heart model, promoting the transition from quiescent fibroblasts to myofibroblasts. An iNOS dependent remodeling in the MI heart *via* peroxynitrite production and subsequent activation of matrix metalloproteinases may be possible.

However, to the best of our knowledge, our study is the first to describe iNOS production in CD206+ and CD163+ macrophages in human MI and healthy control hearts. Crucially, the percentage of CD206+ and CD163+ macrophages expressing iNOS exceeds the percentage of CD68+ macrophages expressing iNOS in the infarcted regions (Figure 3). This presents an interesting extension on the roles of recruited inflammatory vs. resident macrophages in human MI in an iNOS dependent manner.

Additionally, we found increased iNOS mRNA and protein expression in the non-infarcted regions of human MI hearts. Since the supply areas of coronary arteries are not limited to either anterior or posterior wall we assume that an occluded artery causing MI in the anterior wall may also lead to microvascular ischemia in the posterior wall. We believe this to be a likely scenario explaining increased iNOS mRNA and protein expression in the non-infarcted regions, especially in cases with long standing history of coronary artery disease. In a rat infarction model Takimoto et al. (15) also found iNOS mRNA and protein levels upregulated in non-infarcted regions and reported this as long as 56 days post infarction, suggesting a long lasting inflammatory reaction in those non-infarcted regions. These results are in line with the findings from other groups, who reported an increase in macrophage numbers in the non-infarcted area of MI hearts (21, 23). This increase is described as slower but longer lasting than in the infarcted area. Local macrophage renewal additional to blood monocyte recruitment, as well as a secondary inflammation reaction and functions of macrophages in heart remodeling are discussed as reasons for that (21, 23).

In the current study, not only CD68+, but also CD163+ and CD206+ macrophage abundance was increased in the non-infarcted myocardium accompanied by an increased iNOS expression in those macrophages. Besides a distant inflammation following infarction or heart remodeling, which may be a reason for that, macrophages and iNOS are known responders to hypoxia (21). However, Takimoto et al. (15) did not report iNOS protein in control hearts. Furthermore, they reported that cardiomyocytes are the major cells expressing iNOS, especially in the non-infarcted regions. We did not detect any iNOS expression in cardiomyocytes. This may be due to species differences and differences between models of myocardial infarction.

### 4.2. iNOS dependent oxidative stress in human MI hearts

The function of iNOS is widely described as producing NO, which reacts with superoxidanion causing oxidative and nitrosative stress in the myocardium *via* production of peroxynitrite (4), which can be narrowed down by NT stainings, the fingerprint of peroxynitrite in tissue. Still, the extent of iNOS dependent NO production to peroxynitrite in the heart remains unclear, since iNOS is not the only contributor to peroxynitrite production and oxidative stress in the heart (13, 36).

In the current study, strong NT staining was found in MI hearts, in infarcted as well as in non-infarcted areas. These results are in accordance with the results of other studies, which examined the immunohistochemical correlation between iNOS and NT in pathological conditions, such as cardiomyopathies, where NT was described in the cardiomyocytes (36, 37). In our study, NT was mainly detected in the cardiomyocytes, however, we found macrophages being positive for NT. Turillazi et al. (36) suggested an additional mechanism of acute cardiac injury triggered by cocaine, by the increase of iNOS and NT and subsequent oxidative stress in the myocardium of cocaine bodypackers. Feng et al. (13), detected NT staining in the non-infarcted myocardium of mice, which increased moderately with NO production by iNOS. Hence, iNOS increases NT but is not the only contributing/modulating factor. Further, they measured nitrate and nitrite in plasma of MI mice and found a consistent increase with myocardial iNOS expression. In comparison with iNOS^−/−^ mice this increase was significant. This suggests iNOS dependent oxidative stress in MI.

To the best of our knowledge, no distance analysis between iNOS+ cells and NT+ cells in human MI and healthy control hearts has been conducted so far. The results of our study confirm that NT+ cells peak within 10–15 µm of iNOS, suggesting a dependence and a range of activity by iNOS produced NO. Within that perimeter, oxidative damage, such as extracellular matrix remodeling, may appear (9), which may lead to left ventricular remodeling after MI (10).

Shimojo et al. (30) found co-expression of NT and iNOS in cardiomyocytes in postmortem human myocardial infarction hearts, which is not confirmed by our study. Since they did not use any macrophage markers the immunohistochemical results may have not been as enlightening as the results in the current study.

Further, the results of our study indicate that the whole heart is affected by oxidative stress, as hinted by NT expression, and this seems long lasting, as no difference in NT staining between older myocardial infarction and acute myocardial infarction was found. Therefore, an additional mechanism of cardiac injury driven by iNOS and NT production in MI hearts is occuring.

## 5. Conclusion

The results of the current study revealed that macrophages are the main source of iNOS expression in postmortem human MI hearts. However, iNOS production was predominantly detectable in resident CD163+ and CD206+ macrophages, which express typical “M2” macrophage markers. These findings are unexpected given that that iNOS is widely considered a marker of inflammatory “M1” macrophages, at least in mice, and confirms the limitations of the “M1” and “M2” nomenclature used to describe cardiac macrophages.

Furthermore, we found that NT+ cells peak within 10–15 µm distance of iNOS+ cells, indicating that iNOS expression influences the level of NT in human MI hearts, increasing oxidative stress within an activity range of 10–15 µm. The oxidative stress in infarction hearts may be responsible for further damage in the myocardium and an impaired prognosis after MI.

Further studies are needed to describe in detail the role of iNOS expression in cardiac macrophages in human MI and how a prolonged inflammatory reaction involving iNOS expressing macrophages impairs the prognosis after MI. This could lead to improved therapy options.

## 6. Limitations

The myocardial infarction tissue sampled in this study originated from humans with different infarct ages and a heterogeneous regional tissue composition. Therefore, differences within the myocardial infarction group regarding iNOS mRNA and protein expression may be further influenced by the heterogeneous infarction tissues. Since a detailed medical history was rarely available amongst MI and controls, the groups are not risk factor (e.g., diabetes) matched. Additionally, no further information on treatment and clinical characteristics of the MI patients (where clinical treatment was applied) was available. Thus, an influence of medical treatment or the absence of medical treatment, on the iNOS expression in MI hearts cannot be ruled out.

## Data Availability

The original contributions presented in the study are included in the article/Supplementary Materials, further inquiries can be directed to the corresponding author/s.
